# Inhaled siRNA Formulations for Respiratory Diseases: From Basic Research to Clinical Application

**DOI:** 10.3390/pharmaceutics14061193

**Published:** 2022-06-02

**Authors:** Yulin Fan, Zhijun Yang

**Affiliations:** School of Chinese Medicine, Hong Kong Baptist University, 224 Waterloo Rd., Kowloon Tong, Hong Kong, China; fyljdz@163.com

**Keywords:** inhaled siRNA formulations, respiratory diseases, drug delivery barriers, structural modification, delivery systems, clinical application

## Abstract

The development of siRNA technology has provided new opportunities for gene-specific inhibition and knockdown, as well as new ideas for the treatment of disease. Four siRNA drugs have already been approved for marketing. However, the instability of siRNA in vivo makes systemic delivery ineffective. Inhaled siRNA formulations can deliver drugs directly to the lung, showing great potential for treating respiratory diseases. The clinical applications of inhaled siRNA formulations still face challenges because effective delivery of siRNA to the lung requires overcoming the pulmonary and cellular barriers. This paper reviews the research progress for siRNA inhalation formulations for the treatment of various respiratory diseases and summarizes the chemical structural modifications and the various delivery systems for siRNA. Finally, we conclude the latest clinical application research for inhaled siRNA formulations and discuss the potential difficulty in efficient clinical application.

## 1. Introduction

**Figure 1:**
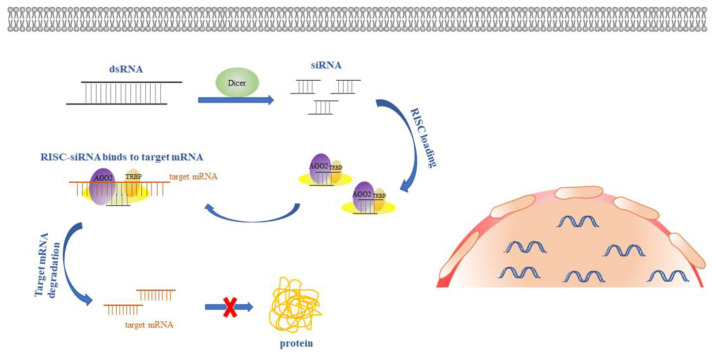
Mechanisms of siRNA-mediated gene silencing.

The concept of RNAi (RNA interference) has emerged with the revelation that double-stranded ribonucleic acid (dsRNA) can induce mRNA degradation. As a natural mechanism, RNAi mediators can regulate target gene silencing in the majority of eukaryotic cells [1]. Small interfering RNA (siRNA), which is a short stretch of dsRNA consisting of 20–25 nucleotides, is the most noticeable candidate among the several varieties of nucleic acids involved in RNAi. Typically, long dsRNA is cleaved into siRNA in the cytoplasm by Dicer. siRNA is then loaded onto the RNA-induced silencing complex (RISC) with associated proteins. The antisense strand (AS) in siRNA binds to the target mRNA via complementary pairing, and then the target gene is cleaved by the AGO2 protein, resulting in gene silencing [2,3] (Figure 1).

In the context of treatment, siRNA operates primarily by antagonizing the target gene. Therefore, to elicit a therapeutic effect, it is sufficient to identify the disease’s causative gene and collect the gene sequence in order to construct a specific siRNA that inhibits the causative gene. Because identifying genes and collecting gene sequences is relatively straightforward and fast, developing siRNA drugs should be easier than small molecule drugs and monoclonal antibody drugs; siRNA drugs should have more precise selectivity, specificity, and potency than conventional chemical drugs. Besides, because siRNA silences genes in the cytoplasm rather than in the nucleus, delivery is comparatively easy [4]. Therefore, as an effective therapeutic approach, relevant research confirms that siRNA can be used in cardiovascular diseases, metabolic diseases, liver diseases, hereditary diseases, cancer and others [5,6]. However, despite the promising theoretical applications of siRNA in drug development, a number of problems limit its clinical implementation. Among these problems are (i) inadequate cellular uptake rate, (ii) structural instability; (iii) enzyme sensitivity, (iv) off-target effects, and (v) proclivity for eliciting immune responses. These constraints have hampered pre-clinical and clinical studies of siRNA [3,7].

Local delivery of siRNA to pulmonary tissue not only allows for non-invasive drug administration but also minimizes drug degradation due to the lung’s low nuclease activity. The large surface area of alveoli can improve drug absorption, reduce the local dose of the drug needed and thereby minimize side effects. Lung epithelial cells are prospective target cells for the treatment of several respiratory diseases, hence inhalation therapy for lung diseases is particularly significant [8,9]. Of course, the therapeutic effect of siRNA inhalation can be influenced by the lung’s anatomy, physiological state, and metabolic features, so special drug delivery systems are required to allow drugs to be deposited in the lungs.

Several articles have reviewed the pulmonary delivery of siRNA which are mainly focused on the challenges of pulmonary delivery and delivery methods of inhaled siRNA [10,11]. As one of the hot issues in the field of nucleic acid drugs, siRNA has been increasingly investigated by researchers in recent years for local delivery. In this review, we introduced the latest research progress on inhaled siRNA formulations for the treatment of lung cancer, respiratory infections and chronic respiratory diseases. We focused on the chemical structure modifications of siRNA and the use of appropriate delivery systems to overcome the delivery barriers of pulmonary administration. We also discussed the latest clinical studies of inhaled siRNA formulations and provided the first overview of research on inhaled siRNA formulations for the treatment of COVID-19.

## 2. Respiratory Diseases Treated with Inhaled siRNA Formulations

### 2.1. Lung Cancer

According to statistics, lung cancer is one of the most prevalent human malignancies [12]. Squamous cell carcinoma, adenocarcinoma, small cell carcinoma, and large cell carcinoma are the histological subtypes of lung cancer [13]. Considering lung cancer development involves a number of genetic and epigenetic alterations, particularly the activation of growth-promoting pathways and the inhibition of tumor-suppressive pathways, it is crucial to regulate the molecules implicated in lung cancer pathogenesis for treatments [14]. By applying siRNA as a therapeutic tool, sequences can be constructed for a specific target, and siRNA can be administered alone or in conjunction with other anti-tumor agents to achieve highly effective and low-toxicity targeted therapy for the inhibition of lung cancer cell growth, invasion, or metastasis.

Non-small cell lung cancer (NSCLC) is the most common type of lung cancer [12]. Currently, chemotherapy and immunotherapy are widely utilized to treat NSCLC; however, these therapies have serious side effects including cytotoxicity, treatment limits, and drug resistance. To mitigate adverse effects and broaden the therapeutic effects, some investigators have combined siRNA targeting EGFR with the anti-cancer drug paclitaxel (TAX) in an inhaled formulation delivery system. The system uses nanostructured lipids (NLC) as carriers and luteinizing hormone-releasing hormone (LHRH) as ligands to target primary and potentially metastatic tumor cells. Their trial demonstrated that siEGFR-TAX-NLC-LHRH significantly improved NSCLC treatment results [15]. siRNA can also be used to inhibit multidrug resistance mechanisms to improve chemotherapy efficacy [16]. Recently, a dry powder inhaler (DPI) preparation for multi-drug resistant lung cancer treatment was developed by integrating lipopolymeric nanoparticles as carriers for the chemotherapeutic drug cisplatin and siRNA targeting ABCC3, a drug efflux promoter [17]. Numerous research findings show that inhaled siRNA formulations offer promising therapy for lung cancer (Table 1).

### 2.2. Respiratory Infection

Respiratory infections, which include viral and bacterial infections, are the most common infectious diseases in the world, with significant morbidity and mortality, and they have a huge economic impact on society [25]. Patients with respiratory viral infections are more likely to develop secondary bacterial infections [26]. Other severe systemic adverse effects also can be caused by some viral infections. Silencing a critical mRNA in the virus or blocking a specific gene in host cells using siRNA, which affects viral replication, can be used to treat respiratory viral infections (Table 2). Bitko et al. [27] demonstrated the benefit of using an intranasal delivery system to convey a specific siRNA to the lung for the prevention and curing of RSV and PIV infections.

Bacterial respiratory illnesses have a significant impact on human lung health and life quality. Tuberculosis, caused by *Mycobacterium tuberculosis* (Mtb) infection, for example, is a severe hazard to public health. However, the current therapeutic technique has a long cycle. Serious toxic side effects and drug resistance can occur. Consequently, more effective treatments are urgently required [28]. Studies have shown that human lymphotactin (XCL1) can increase the granulomatous reaction induced by Mtb infection. Relying on this finding, siXCL1 was designed and administered via aerosols. Results suggest that local targeted delivery of siXCL1 decreased the expression of XCL1 during Mtb infection and reduced the granulomatous and fibrotic response in the lung, which is beneficial to tuberculosis treatment [29]. Pneumonia is another serious, prevalent clinical respiratory disease. Severe pneumonia can result in acute lung injury, acute respiratory distress syndrome, cytokine storm, and an increased risk of death. TNF-α has been proven to play a crucial role in the development of a cytokine storm. In order to transmit siTNF-α, nanocomplexes (NCs) are chosen as carriers, and the system is delivered by intratracheal administration. Results indicate that siTNF-α-NCs can penetrate the mucus layer efficiently, and the treatment is remarkably successful in severe pneumonia [30] (Table 2).

**Table 2:** Research of inhaled siRNA formulations for respiratory infections.

Disease	Target	Administration	Delivery System	Ref.
Influenza	Nucleoprotein	Intranasal administration	Chitosan nanoparticles	[31]
Influenza	Nucleoprotein, acidic polymerase	Intranasal administration	Oligofectamine	[32]
H1N1	Nucleoprotein, acidic polymerase	Inhalation	PH-responsive peptides	[33]
RSV	N-protein	Intranasal administration	Naked siRNA	[34]
RSV	RSV-protein	Intranasal administration	Naked siRNA	[35]
RSV	NSP1	Intranasal administration	Chitosan nanoparticles	[36]
Pneumonia	TNF-α	Intratracheal delivery	RC-NCs	[30]
Tuberculosis	TGFβ1	Inhalation	Naked siRNA	[37]
Tuberculosis	XCL1	Oro-tracheal administration	Naked siRNA	[29]

Notes: RC, an inflammation-sheddable, charge-reversal pro-peptide of RAGE-binding peptide (RBP); NCs, nanocomplexes.

### 2.3. Chronic Respiratory Disease

Chronic respiratory diseases (CRDs) are a widespread set of disorders associated with a high rate of morbidity and mortality globally. They include asthma, bronchiectasis, chronic obstructive pulmonary disease (COPD), and interstitial lung diseases (ILDs) [38]. Asthma can be defined as an allergic disease to some extent, so inhaled glucocorticoids and β2-receptor agonists are commonly used to ease symptoms. However, these treatments are ineffective for certain people with severe asthma. New therapeutic alternatives are still required. Because multiple inflammatory factors are involved in the inflammatory process in asthma, modulating key pro-inflammatory genes may result in asthma improvement [39], and several research findings corroborate this possibility (Table 3). GATA3, a transcription factor that regulates the differentiation of T cells into TH2 cells, is an important upstream factor in the disease process of asthma and is, therefore, presumed to be an effective therapeutic target. Eventually, the siGATA3-transferrin (Tf)-melittin (Mel)-polyethyleneimine (PEI) complex was established after extensive screening and research on the complex carriers. Currently, research is exploring combining prepared formulas with powder inhaler technology in order to develop new asthma treatment options [40].

COPD is another multifactorial complicated respiratory disease. Together with the clinical symptoms of cough and dyspnea, some patients may suffer systemic inflammatory responses, and a lack of appropriate treatment can worsen their condition [39,41,42]. Various inflammation-related components can be targeted to improve the effectiveness of therapies and reduce clinical symptoms. Cytokines are one such target, and siRNA nanoparticles that inhibit cytokine signaling can be delivered to pulmonary tissues to lower inflammatory effects and reduce symptoms associated with lung illness [41]. Numerous studies have shown that developing siRNAs with specific sequences for inflammation-related components for pulmonary delivery can be employed to treat COPD’s inflammatory response (Table 3).

**Table 3:** Research of inhaled siRNA formulations for chronic respiratory diseases.

Disease	Target	Administration	Delivery System	Ref.
Asthma	GATA3	Inhalation	Tf-Mel-PEI	[40]
Asthma	Chil3, Chil4	Intratracheal Administration	HMG-OR	[43]
Asthma	VDBP	Intra-tracheal instillation	DEXA-PEI	[44]
Asthma	c-Kit	Intranasal administration	Modified siRNA	[45]
Asthma	SOCS3	Intranasal administration	Naked siRNA	[46]
Asthma	Syk	Intranasal administration	Naked siRNA	[47]
COPD	RIP2	Intratracheal administration	Naked siRNA	[48]
COPD	RPS3	Intratracheal administration	Naked siRNA	[49]
COPD/ILD	Cytokine Signaling	Nasal instillation	CaP-PLGA	[41]
COPD	MAP3K19	Intratracheal administration	Naked siRNA	[50]

Notes: Tf, transferrin; Mel, melittin; PEI, polyethyleneimine; HMG, high mobility group; OR, oligoarginine; DEXA-PEI, dexamethasone-conjugated polyethyleneimine; CaP, calcium phosphate nanoparticles; PLGA, poly(lactic-co-glycolic acid).

## 3. Inhaled siRNA Barriers

**Figure 2:**
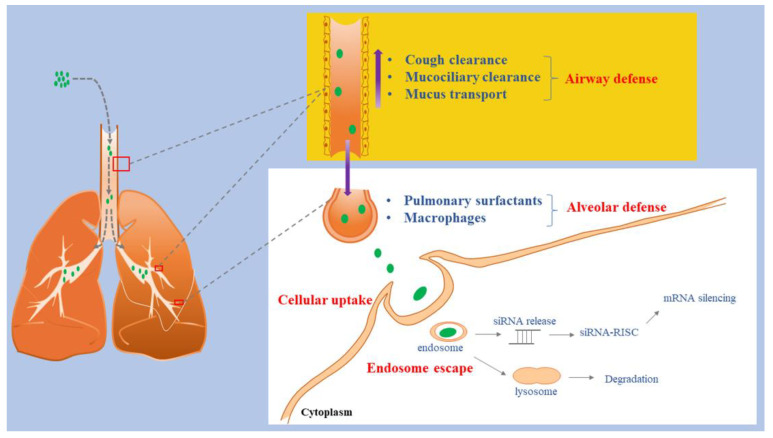
Schematic illustration of siRNA inhalations delivery barriers.

Once they enter the blood circulation system, siRNA delivered systemically are likely to be degraded by the abundant endonucleases in the serum. Because siRNAs are negatively charged and have large molecular weights and poor stability, the half-life of siRNA is short. In theory, siRNA pulmonary inhalation has significant clinical therapeutic advantages compared to systemic administration [51]; however, in practice, there are significant challenges (Figure 2).

### 3.1. Pulmonary Barriers

#### 3.1.1. Airway Defense

The respiratory system can be functionally divided into conducting airways and respiratory regions. Conducting airways do not perform gas exchange, whereas the respiratory region, which is composed of respiratory bronchioles, alveolus pulmonis and alveoli, performs the function of gas exchange. The branching structure of the airways facilitates inertial impaction, deposit, diffusion, and retention of inhaled particles, providing a screening mechanism for drug molecules. The pattern of molecule deposition in pulmonary tissue is highly influenced by the aerodynamic diameters of particles. Generally, particles with an aerodynamic diameter > 5 µm are mainly deposited in the upper airways, particles with an aerodynamic diameter of 1–5 µm are mostly transported to the lower respiratory tract for deep lung deposition, while particles with an aerodynamic diameter < 1 µm are largely exhaled. As for particles with an aerodynamic diameter less than 100 nm, i.e., nanoparticles, about 50% are deposited as aggregates in the alveolar region. For this reason, the delivery region must be determined based on the therapeutic purpose and disease condition, and the particles should be designed with an appropriate diameter for targeting that region [51,52,53].

Mucociliary clearance (MCC) is an essential defense mechanism against inhaled irritants and excessive airway secretions. MCC is comprised of the mucous blanket, ciliated columnar epithelial cells, cilia, goblet cells and serous glands. The cilia in the airway all move in the same direction, propelling the mucus and any trapped particles to the throat, where it is swallowed or coughed out [54]. Mucus is mainly composed of water, mucin, free protein, inorganic salts, and lipids. Besides water, the main macromolecule is mucin, which has a negatively charged O-glycosylation structure and a protein backbone. siRNA, which is likewise negatively charged by inhalation delivery, will naturally accumulate in mucus and be promptly removed from the respiratory tract due to electrostatic action. To avoid this, a suitable vector for lung delivery of siRNA is essential. Additionally, mucin can form a macromolecular network on the airway surface, increasing the viscosity of the epithelial cell surface. If the drug particle is too large, it cannot pass through the reticular pores of the mucus, reducing the drug’s penetration and diffusion rates [55]. Small particles can simply traverse mucus, escape MCC, and reach epithelial cells [56].

Coughing, another essential airway defense mechanism and one of the main symptoms of respiratory disease, supports the removal of foreign substances and excessive secretions that enter the respiratory system. Coughing can not only change the pleural pressure but also the velocity of airflow in the respiratory tract, thus affecting particle dispersion in pulmonary tissue [57]. For example, approximately 60% of accumulated particles can be eliminated by coughing in patients with COPD [58]. In administering therapeutic formulations, factors including drug particle morphology, pH value, solubility, drug flow rate, molecular state, and dosage can affect coughing. In summary, coughing is influenced by the patient’s physiological state and formulation’s characteristics during pulmonary administration. It is important to note that there are no cough receptors in the alveoli. Therefore, any fine or ultrafine particles deposited in the alveoli will not cause cough, whereas coarse particles with a particle size > 5 μm will be deposited in the upper respiratory tract by airflow, causing irritation [57]. Thus, choosing a suitable siRNA particle size can minimize host irritation and coughing, and significantly increase the particle deposition in the lung.

#### 3.1.2. Alveolar Defense

Alveoli are hemispheric vesicles formed from a single epithelial cell layer; they are the main site of gas exchange. The alveolar surface is covered by a thin layer of pulmonary surfactant (PS). PS is a complex blend of lipids (90%) and proteins (10%). The lipid components are mostly composed of phospholipids and glycerol, which are necessary for the maintenance of alveolar surface tension. Proteins are classified into four types: SP-A, SP-B, SP-C and SP-D. SP-A and SP-D are hydrophilic proteins that deliver pathogens and particles to immune cells, they are critical components of the innate immune system of the lungs. In contrast, SP-B and SP-C are hydrophobic proteins that reduce alveolar surface tension, prevent alveolar collapse and maintain normal respiratory function when bound to phospholipids [59]. While PS is required for host respiration, defense, and survival, it is one of the main obstacles to entry into pulmonary tissue for siRNA inhaled formulations. The physicochemical and biological features of PS give it many advantages when used as a carrier for pulmonary drug delivery, including protection of the drug as it crosses into lung tissue, increased efficiency of drug delivery in the airway and lung, and dissolution of insoluble drugs. Previous research has found that nanodrugs administered via the lungs frequently fail to overcome the extracellular barrier. Combining PS with nanoparticles into a single polymeric delivery system could enhance siRNA distribution [60].

Alveolar macrophages (AMs) are positioned on the luminal surface of the alveoli and are the primary phagocytic cells capable of defending against respiratory infections in the distal lung tissue, accounting for more than 90% of total lung immune cells. Through phagocytosis and the secretion of lysozyme, protease, and antimicrobial peptides, AMs eliminate microscopic materials inhaled daily by the host, with the best phagocytosis for particles 1.5–3 μm in size [56]. When a large number of microbes or particles are present in the respiratory system, however, AMs secrete a range of cytokines, chemokines, and arachidonic acid metabolites that trigger an inflammatory response [61,62]. Simultaneously, neutrophils accumulate at the infection site to amplify the anti-inflammatory action of macrophages when they first encounter a pathogen [53]. It implies that AMs are associated with high gene expression in inflammatory lung lesions and could be targeted with siRNA therapy in inflammatory lung disease. Due to these functional characteristics, delivering siRNA to the target site is challenging.

Enzymes, such as proteases and ribonucleases are also major variables that affect siRNA effectiveness. Last but not least, disease conditions can alter the respiratory system’s environment, such as the quantity, viscosity, and composition of mucus in the airways, which can in turn affect the efficiency of siRNA-targeted lung drug delivery [53].

### 3.2. Cell Barriers

After evading lung clearance, siRNA must pass the cell membrane and function in the target cell’s cytoplasm. However, since siRNAs are negatively charged hydrophilic macromolecules that do not effectively cross biological membranes, the challenge of siRNA target delivery after crossing the lung barrier can be separated into two components [63]: (i) To facilitate effective siRNA internalization via cellular uptake, siRNA is typically complexed with positively charged liposomes, polymers, and/or other carriers to form positive nanoparticles. These positive nanoparticles can bind to negatively charged cell membranes via endocytosis or can conjugate with ligands that recognize specific antigens on the surface of target cells to complete cellular internalization [64]. (ii) Intracellular transfer of siRNA is from the early endosome to the late endosome, followed by the development of lysosomes containing RNases to destroy siRNA. In order to avoid being trapped and destroyed, siRNA must be released from the endosome into the cytoplasm prior to degradation [64]. Owing to the low acidity of lysosomes, using acid-responsive delivery vehicles to enable siRNA release is an ideal technique for circumventing cellular barriers [65].

## 4. siRNA Structural Modification

As demonstrated by the preceding discussion, while siRNA has promising development and utilization prospects, there are still numerous hurdles to overcome on the path from the laboratory to the clinic. To achieve a therapeutic effect, extracellular and intracellular barriers must be overcome. To maximize the therapeutic effect while reducing or avoiding adverse effects, the siRNA structure can be modified, or alternative delivery systems can be devised. This section focuses on the various types of chemical structural modifications of siRNA. Depending on the site of the structural modification, chemical modifications can generally be classified as ribose, base and backbone modifications [3].

### 4.1. Ribose Modification

**Figure 3:**
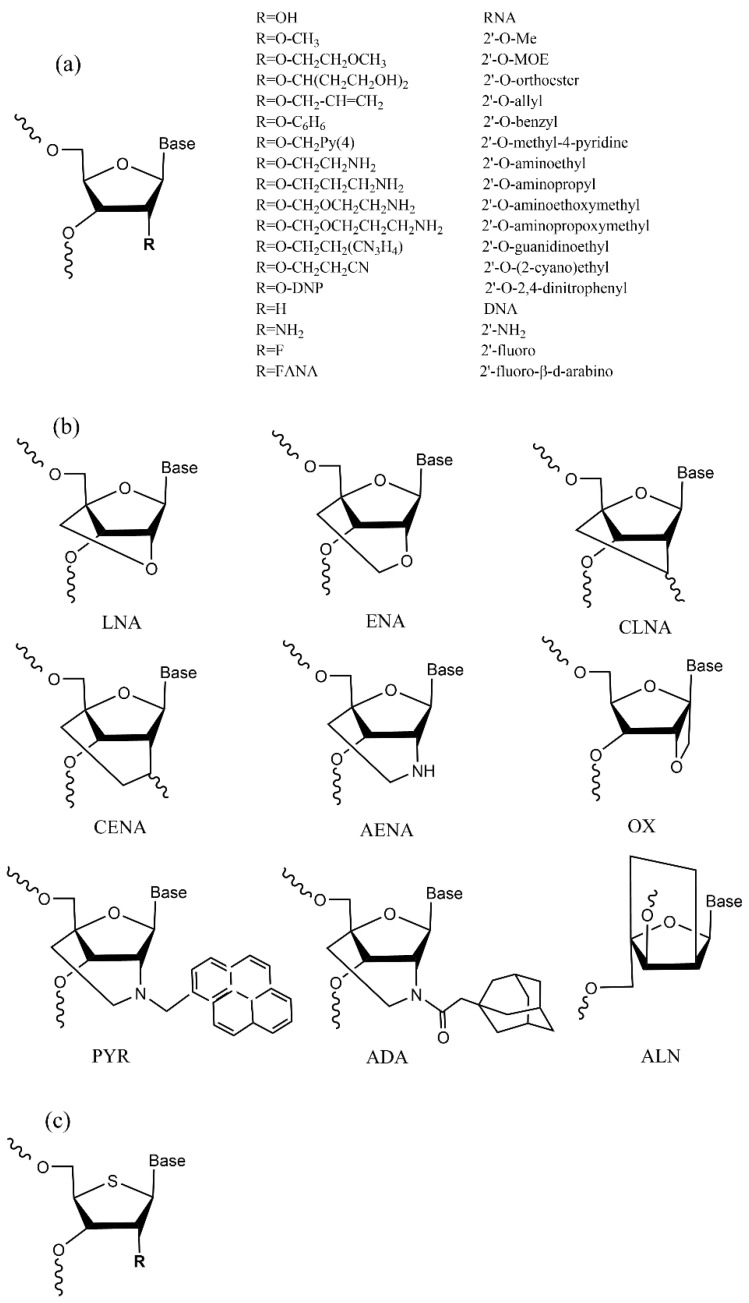
Chemical modification types of siRNA ribose. (**a**) 2′ position modifications of siRNA ribose; (**b**) Isomeric modifications of siRNA ribose. LNA, locked nucleic acid. ENA, ethyl-bridged nucleic acid. CLNA, 2′, 4′-carbocyclic-LNA-locked nucleic acid. CENA, 2′, 4′-carbocyclic-ENA-locked nucleic acid. AENA, 2′-deoxy-2′-N,4′-C-ethylene-LNA. OX, oxetane-LNA. PYR, 2′-N-pyren-1-ylmethyl-2′-amino-LNA. ADA, 2′-N-adamantylmethylcarbonyl-2′-amino-LNA. ALN, a-L-LNA; (**c**) 4′-S modification of siRNA ribose.

siRNA ribose modification is the most frequently used and mature method for the chemical modification of ribonucleic acid. Since 2′-OH attacks the phosphate group first when RNA is hydrolyzed, modifying the 2′ position substantially improves the stability and half-life of siRNA [3,66,67,68] (Figure 3a). Data from a study suggest that [69] 2′-OMe-modified siRNA is more stable in vivo and has a higher affinity for target mRNA, and the modification is more tolerated in AS’s center position than at the 3′-end or 5′-end. Interestingly, siRNA with 2′-OMe in the sense strand (SS) is unlikely to be affected by the substitution position. One investigation indicated that modifying 2′-OMe at position 2 of the AS can potentially reduce off-targeting [70]. Similar to 2′-OMe, 2′-OMOE modified at the 3′- or 5′-end of AS is significantly less active than when it is modified in the central position, particularly at the 5′-end of substitution. However, 2′-OMOE modified in SS exhibits better activity and is unaffected by the substitution position. These results suggest that the 5′-end of the AS may be the recognition site for the helicase. Therefore, the group with a larger steric volume will affect the binding of the helicase to the recognition site, which is negatively correlated with the siRNA activity. Notably, siRNA tolerates groups with a higher steric volume than typical modifications, such as 2′-O-benzyl and 2′-O-methyl-4-pyridine. These modifications enhance the efficacy and duration of siRNA in vivo [71]. Another set of research concluded that [72] increasing the 2′-O cationic group stabilized the double strand of the siRNA molecule, and that the 2′-O-aminoethyl modified siRNA was the most stable and active when compared to 2′-O-guanidinoethyl, 2′-O-(2-cyano)ethyl, and 2′-O-allyl modified siRNA. Two distinct conformations, 2′-F and 2′-FANA, were well tolerated and increased the in vivo stability of siRNA duplexes on both the SS and AS [73,74].

Isomeric modification is another way to modify ribose [75,76] (Figure 3b). LNA (locked nucleic acids) is a single carbon bridge formed by linking the 2′ and 4′ of the sugar ring with a methyl group. This rigid structure improves the stability of the nucleotide and its affinity for the target gene [77]. LNA has been found to mitigate siRNA’s off-target effect by inhibiting target mRNA cleavage by the SS [78]. However, applying LNA modifications to the AS at positions 10, 12, and 14 may inactivate the siRNA, and using excessive amounts of LNA-modified siRNA in vivo may cause hepatotoxicity [79]. ENA (ethylene bridge nucleic acids) has a double carbon bridge structure, the same as LNA, which improves the thermodynamic stability of RNA. A comparison of ENA’s in vivo activity to that of 2′-OMe modification revealed that ENA was more effective than 2′-OMe modification, presumably due to ENA’s higher affinity for the target gene [80]. However, findings indicate that ENA modified at either the 3′-end or 5′-end of the strand decreases the effectiveness of siRNA [67]. The success of LNA and ENA modifications has also inspired the exploration of 2′-O. Some scholars have replaced O with the less electronegative C, and this substitution was found to reduce siRNA’s affinity for binding to mRNA [81].

Another modifiable position for siRNA is the 4′-thio-modified nucleotide, which refers to the substitution of a sulfur atom for an oxygen atom in the sugar ring (Figure 3c). Because S has a lower electronegativity than O, the C-S bond is longer than the C-O bond. The chemical characteristics of the C-N glycosidic bond are also affected to a certain extent, whereas the base is not considerably affected. These characteristics make the modification of 4′-S beneficial to nuclease with higher stability. When 4′-S is combined with 2′-ribose modification, siRNA can show enhanced activity and stability [82,83]. However, the activity is influenced by the 4′-S modification site and quantity in AS. The central position is particularly vulnerable to modification, whereas the SS has a high tolerance for 4′-S modification [83]. Under certain circumstances, siRNAs in which both strands have been modified with 4′-S are more active than those modified on only one strand [84].

### 4.2. Phosphate Backbone Modification

**Figure 4:**
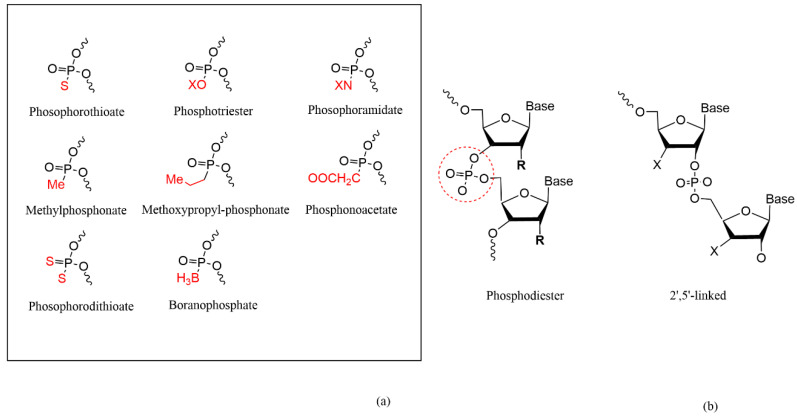
Chemical modification types of siRNA phosphate linkage. (**a**) The modifications of phosphodiester bond; (**b**) The modification of 2′,5′-phosphodiester linkage.

While the nucleotide backbone is critical for the clinical therapeutic role of oligonucleotides, the phosphodiester (PO) bond in natural nucleic acids is frequently hydrolyzed by nucleases, thereby limiting their therapeutic utility in vivo. Thus, the phosphate backbone serves as a structural modification site for siRNA [3,70,81] (Figure 4). Phosphorothioate (PS) incorporation is a very common method of modifying the phosphate backbone of oligonucleotides and is widely applied in antisense oligonucleotide (ASO) technologies to enhance antinuclease activity and plasma protein binding of oligonucleotides. Due to the negative charge of PS, it lowers the Tm value of the RNA double strand. Appropriate PS modifications can improve the metabolic stability of siRNA; however, excessive PS modifications reduce siRNA activity and increase the potential for toxic side effects due to the non-specific binding of PS to certain cationic proteins [70,85]. PS contains a chiral atom, P, which forms two isomers, Rp and Sp. These two isomers have different physicochemical properties and have a substantial impact on siRNA. The quantity of Rp configurations of PS in the center of SS is proportional to the potency of siRNA, which can increase the binding of siRNA to RISC. Because the Sp conformation is more nuclease stable, Sp-PS modifications can reduce the cleavage of the SS [86]. Phosphorodithioate (PS2) is a phosphodiester structure in which both the non-bridging oxygen atoms are replaced with sulfur atoms. PS2-modified siRNA has strong resistance to nucleases and also possesses position-dependent gene silencing activity. The Tm value of the PS2-modified double-strand is slightly reduced, and the introduction of PS2 modifications at the 3′-end of the SS facilitates double-strand unwinding and enhances siRNA activity in vivo [87].

Not only is PO rapidly destroyed by nucleases, but its negative charge also results in poor cell membrane permeability for siRNA, making it difficult for cells to take up. Thus, the neutral phosphotriester skeleton can be employed, which can be transformed to PO in the cytoplasm by thioesterase [88]. Moreover, a study has demonstrated that incorporating the phosphotriester backbone at the 3′-end of the SS might enhance siRNA’s resistance to nucleases [89]. Methylphosphonate (MP) modification is the substitution of a non-bridging oxygen atom in PO with a methyl group, which is also a neutral structure that enhances cell membrane uptake and is highly resistant to nucleases. MP is a chiral structure that exists in two conformations, Rp and Sp, with the Rp conformation possessing a higher affinity for binding to mRNA [81]. Phosphonoacetate-modified phosphate backbones render siRNA nuclease-resistant due to the presence of P-C bonds [90]. According to some researchers, another way for increasing the biological stability of nucleic acid is boranephosphate modification, in which the nuclease resistance is more than ten times that of unmodified siRNA. While the boranephosphate bond remains negatively charged, the charge distribution is different from that of PO and PS, increasing the molecule’s hydrophobicity. However, boranephosphate incorporation is difficult to obtain chemically, and the modification at the center of AS limits siRNA activity [91].

Esterification of neighboring nucleotide 2′-OH and 5′-phosphate groups to form 2′,5′-phosphodiester linkage is another kind of modification that possesses anti-enzymatic properties. Data have demonstrated that the appropriate 2′,5′-phosphodiester linkages in SS promote the loading of the AS onto RISC to stimulate activity; therefore, SS is more tolerant to this modification than AS [92]. Except in exceptional circumstances, increasing the number of 2′,5′-phosphodiester bonds reduces siRNA activity, which may be attributed to less siRNA binding to the AGO2 protein. Another study discovered that siRNAs containing a mixture of 2′,5′/3′,5′-phosphodiester bonds on the AS were more effective at silencing genes than siRNAs containing only 2′,5′-phosphodiester bond modifications [93].

### 4.3. Base Modification

**Figure 5:**
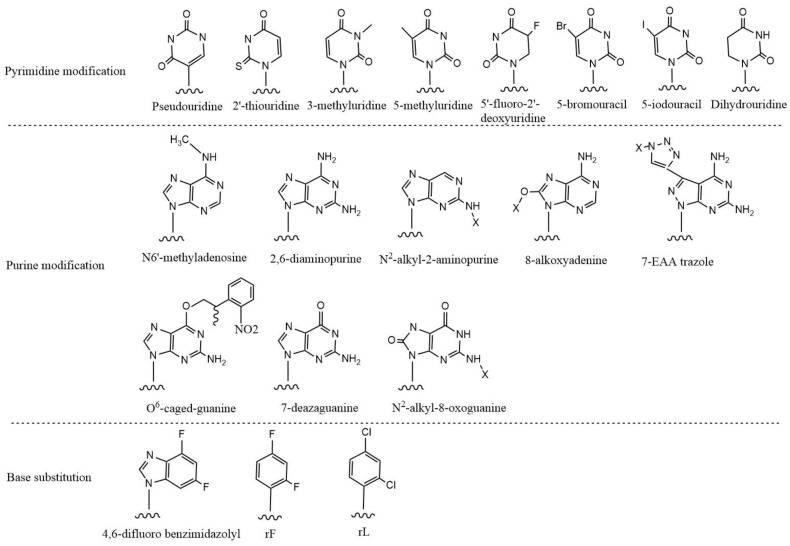
Chemical modification types of siRNA base.

Base modification is the chemical modification of siRNA’s base groups and is categorized as pyrimidine modification, purine modification, or base replacement [3,66,76,94] (Figure 5). Base modification is less prevalent than sugar ring and phosphate backbone modification, but it is also a method for regulating double-strand interactions, assisting AS in binding to RISC and suppressing immunological responses [76]. Pseudouridine is a ribose isomer of uridine and is one of the earliest recognized RNA structural modifications. Although the complimentary base pairing is unaffected, the N-C bond between the base and ribose is converted to a C-C bond, increasing the stability of the RNA structure [95]. By incorporating a 2′-thiouridine structure into the double strand, siRNA becomes more hydrophobic and hence more readily accepted by cells. Additionally, the S atom at the 2-position strengthens the base stacking force, increasing the affinity of the siRNA for the target gene. The thermodynamic stability and gene silencing activity of siRNA containing pseudouridine, 2′-thioureidine and dihydrouridine were evaluated. Results showed that dihydrouridine modification in the central part of the AS significantly reduced double-strand stability, probably due to disruption of base stacking by non-aromatic bases. In contrast, pseudouridine and 2′-thioureidine modification in the central part maintained the stability of the double strand. The most positive effect of these three modifications was found to come from the modification of 2′-thioureidine at the 3′-end of the SS, combined with the modification of dihydrouridine at the 5′-end of the SS. These two modifications increased activity by 25–50% [96]. This implies that the simultaneous modification of bases at both ends of the siRNA strand increases its thermodynamic asymmetry, which is advantageous for RISC binding to the AS. The introduction of 5-methyluridine into the AS increases the in vivo stability and activity of siRNA, while the introduction of 3-methyluridine affects hydrogen bonding at the cleavage site in AS, thereby reducing the effect of siRNA [79].

N6′-methyladenosine (m6A) is a naturally modified nucleoside that contains a methyl group at the adenosine N6 position. Replacing all adenosine with m6A can diminish the immunological response generated by siRNA, whether on AS or SS [97]. The addition of 5-bromouracil, 5-iodouracil, and 2,6-diaminopurine increases the stability of A-base pairing and the affinity of siRNA for the target mRNA, but inhibits siRNA double-strand unwinding and thus inhibits RNAi efficacy [98]. The Tm value of siRNA decreases when 2,4-difluorobenzene (rF) and 2,4-difluorochlorine (rL) are substituted for U, but modified siRNA still maintains substantial gene repression activity, owing to the structural similarity of rF and rL to U [99].

## 5. siRNA Inhalation Delivery Systems

siRNA can be structurally modified to improve, to some extent, double-strand stability, reduce immunological stimulation and off-target effects, and increase gene silencing activity. However, siRNA delivered by inhalation without a carrier may fail to reach the target location. An appropriate delivery system can be used to ensure siRNA’s therapeutic efficacy. The optimum siRNA delivery vehicle supports siRNA in crossing the lung and cellular barriers without impairing its function, shields siRNA from degradation, allows siRNA endosome escape, and is non-toxic and biodegradable [100].

### 5.1. Lipid Nanoparticles

**Figure 6:**
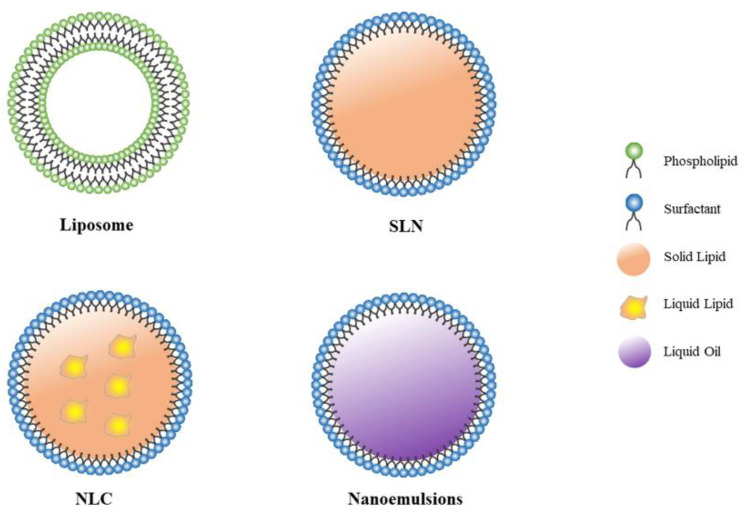
Types of LNPs used for siRNA pulmonary delivery.

Lipid nanoparticles (LNPs) are a category of biocompatible, multicomponent lipid systems that can be implemented as delivery vehicles to enhance the stability and efficacy of drugs in vivo while also minimizing adverse side effects [3] (Figure 6).

#### 5.1.1. Liposomes

LPNs are most commonly represented by liposomes. Cationic liposomes (CL) can form a complex with negatively charged siRNA through electrostatic interaction to form CL-siRNA, which can avoid degradation by RNase and promote endocytic uptake. The two key parameters affecting its transfection efficiency (TE) are the membrane charge density σ_M_ of the cationic liposome membrane and the charge ratio ρ_chg_ of the cationic lipid to nucleic acid (NA). It was found that a high σ_M_ value is required for CL-NA to escape from the inner vesicle, but a low σ_M_ value is required for dissociation of the complex in the cytoplasm after the escape. In other words, the σ_M_ value must be carefully adjusted for maximum TE. Additionally, considering that siRNA has a relatively short duplex length, the electrostatic stability of the complex formed with CL is low, requiring a higher ρ_chg_ to achieve efficient siRNA delivery. Therefore, cationic multivalent lipids (MVL) are superior carriers for siRNA than univalent lipids (UVL) [101].

Although CL is an effective delivery system, it can cause an inflammatory response in vivo and is potentially cytotoxic, which is related to factors, such as the chemical structure and concentration of CL [100]. PEG is frequently used to modify CL, hence minimizing the harmful effects generated by CL [11]. Coating CL with the natural anionic polymer hyaluronic acid (HA) significantly reduces cytotoxicity and preserves the silencing effect of siRNA [102], and in the preparation of spray-freeze-dried particles (SFDPs) for pulmonary drug delivery, it was found that the RNAi effect of CL carriers coated with HA was significantly better than that of CL carriers not coated with HA [103]. An investigation has shown that complementing LNPs with appropriate auxiliary lipids can improve delivery efficiency and stability while minimizing adverse effects. The commonly used auxiliary lipids are mostly neutral lipids, such as CHOL, DOPC, DOPE [104]. Shim et al. [22] screened six nanoliposomes and discovered that ECL, a nanolipid complex composed of EDOPC-CHOL-DOPE (8:5:2), had the highest in vivo delivery efficiency, and intratracheal administration of ECL-Mcl 1 siRNA significantly inhibited Mcl 1 expression and suppressed metastatic lung cancer growth in mice. Another significant advancement is the use of ionizable lipids. The most frequently used ionizable lipids are DLin-MC3-DMA and DLin-KC2-DMA [105]. These lipids are pH sensitive and are electrically neutral. At pH 7.0, they ensure the stability of LNPs-siRNA and reduce the toxic effects caused by excessive positive charges, while under acidic conditions in lysosomes they are protonated and interact with the anionic phospholipid membrane, disrupting the membrane structure and thus releasing siRNA [106].

#### 5.1.2. Solid Lipid Nanoparticles

Solid lipid nanoparticles (SLNs) are solid lipid-based drug delivery systems that encapsulate one or more bioactive pharmaceuticals in a lipid-like core. They have high transfection rates, low cytotoxicity and can prolong the in vivo half-lives of drugs, making them commonly used as delivery vehicles for siRNA [107]. However, SLNs have the disadvantages of low drug loading and instability. Hanafy et al. [108] recently encapsulated PD-1 siRNA in SLN and discovered the encapsulation rate reached 98.9% following physical characterization. In vitro and in vivo research demonstrated that siRNA targeting PD-1 can greatly decrease tumor growth. To enable SLNs-siRNA-targeted pulmonary drug delivery, some scholars used thin-film freeze-drying (TFFD) to prepare SLNs-TNFα siRNA dry powder formulations, in which SLNs were composed of lecithin, cholesterol and stearic acid-polyethylene glycol conjugates. Their tests showed that the TFFD technology could maintain the particle size, PDI, zeta potential and gene silencing effect of dry powder particles in vitro. The result of the in vitro simulated penetration of the mucus layer showed that the SLNs dry powder effectively penetrated the mucus layer. Probably it was the PEG modification in the SLNs carrier and the weaker negative zeta potential property that facilitated the penetration of the particles into the mucus layer. From this, researchers tentatively concluded that SLNs dry powder could be deposited into the lung [109].

#### 5.1.3. Nanostructured Lipid Carriers

Nanostructured lipid carriers (NLCs) are novel nanocarriers developed on the basis of SLNs, which are composed of a mixture of solid and liquid lipids in different proportions and have higher drug loading efficiency than SLNs. Despite their certain cytotoxic effects and the irritating and sensitizing effects of surfactants, NLCs are still well suited for delivering drugs with a variety of physicochemical properties [110]. Besides, NLCs can improve bioavailability, biocompatibility, and slow-controlled release capability [111,112]. NLC inhalation formulations have been the subject of numerous investigations and applications [113,114,115]. Taratula et al. [20] created an effective inhalation preparation of NLCs-siRNA that incorporated MRP1 siRNA and BCL2 siRNA, as well as the anticancer drug TAX. The surface of NLCs was modified with LHRH analog peptides corresponding to those overexpressed receptors on the membrane of lung cancer cells as target sites to achieve drug-targeting effects. In vivo experiments have demonstrated that NLCs-siRNA performs well in local delivery to the lungs, effectively suppressing tumors while having no apparent detrimental effects on healthy organs.

#### 5.1.4. Nanoemulsions

Nanoemulsions are submicron colloidal particle systems composed of a water phase, oil phase and emulsifier; they can be divided into three categories: O/W, W/O and multiple nanoemulsions. As a drug carrier, nanoemulsions have the advantages of being non-toxic and non-irritating and can improve the stability of the drug. Their large surface area promotes drug absorption and improves the bioavailability of the drug [116,117]. Some researchers have applied nanoemulsions in pulmonary drug delivery devices because of these physical and chemical advantages. Metastatic lung cancer is a common and fatal clinical disease. CXCR4 and STAT3 are critical regulators of cancer cell metastasis and invasion. Perfluorocarbon (PFC) nanoemulsions are endocytosis-enhancing nanoemulsions with a continuous perfluorocarbon phase and a discontinuous aqueous phase that are kinetically stable and often used for drug delivery and bioimaging. Refs. [23,118] developed a fluorinated polymer CXCR4 antagonist FM, and STAT3 siRNA using PFC as a carrier. FM was used as a surface stabilizer to cover the PFC to form a stable and positively charged FM@PFC drug delivery system. The FM@PFC-siSTAT3 nanoemulsion formulation was finally prepared after siSTAT3 encapsulation in PFC. In vivo studies confirmed that the pulmonary administration of FM@PFC-siSTAT3 concentrated the drug in the lung, resulting in a significant reduction in the number and size of lung metastases, improved anti-cancer activity, and prolonged survival in mice when compared to intravenous administration. LD et al.’s development of PAMD@PFOB/siSTAT3 further established the viability and clinical utility of nanoemulsions for pulmonary administration [119]. However, the use of nanoemulsions as a carrier has to overcome the following points: (i) expensive preparation process, (ii) less selectivity of applicable excipients, and (iii) instability during storage [120].

### 5.2. Polymeric Nanoparticles

**Figure 7:**
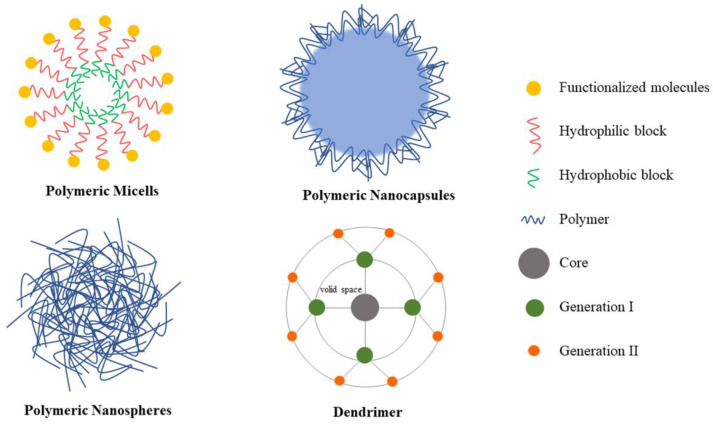
Types of polymer nanoparticles used for siRNA pulmonary delivery.

Polymers are a class of modified drug delivery systems that are simple to prepare, biocompatible, biodegradable and non-toxic, and cause little or no immune response in vivo; they are commonly used as carriers for siRNA delivery [121] (Figure 7).

#### 5.2.1. Polymeric Micelles

Polymeric micelles (PM) are nanostructures with a hydrophilic shell and hydrophobic core formed by the self-assembly of amphiphilic copolymers with high drug loading capacity and stability, and also are potential vehicles for pulmonary drug delivery [122]. Commonly used hydrophilic block materials include polyethylene glycol (PEG), polyamino acids (PAA), polyvinyl alcohol (PVA), and polysaccharides. The mainly used hydrophobic materials include polyglycolic acid (PGA), polycaprolactone (PCL), polypropylene oxide (PPO), and poly-L-histidine (pHis) [123]. Hydrophilic groups can prolong the biological half-life of siRNA and coupling them with a variety of ligands can increase target function and action even further. There are two strategies for forming siRNA polymer micelles [123,124]: (i) Binding of hydrophilic or hydrophobic groups to siRNA followed by condensation with polycations to form micellar structures, which are called polyion complex micelles (PIC) or polyelectrolyte complex (PEC) micelles. PIC protects siRNA from hydrolysis by in vivo enzymes and the commonly available polycationic segment is conventionally a PAA, e.g., PEI. (ii) Micellarizing the complexations that consist of siRNA and polycations containing amphiphilic block copolymers. Yoon et al. [125] linked hydrophilic PEG and hydrophobic lipids to the 3′ and 5′ ends of the siRNA SS, respectively, such that the siRNA spontaneously generated SAMiRNA nanospheres with a hydrophobic center and a hydrophilic outer shell. In comparison to lipid carriers, SAMiRNA induces little immune response, and intratracheal administration can efficiently inhibit target gene expression and treat lung diseases. Subsequently, Choi et al. [43] developed the HMG–Chil3/Chil4 siRNA-OR complex to treat asthma. The high mobility family (HMG), a ligand for TLR4 and RAGE on the surface of AMs, is positively charged and can be targeted by binding to siRNA through electrostatic interaction. Oligoarginine (OR), which consists of three arginine residues and six valine residues in aqueous solution to form positively charged micelles, can act as a carrier for HMG-siRNA complexes. Chil3/Chil4 is mainly produced by AMs during inflammation and is a potential therapeutic target for allergic asthma. Studies have demonstrated that HMG-siRNA-OR can be specifically delivered to AMs by intratracheal administration to suppress asthma symptoms and reduce inflammation. However, for the better application of polymeric micelles as carriers, the drug incorporation and polymer synthesis technologies need to be improved [126].

#### 5.2.2. Polymer Nanocapsules

Polymer nanocapsules (NCs) are a new technology for delivering siRNA consisting of a polymer shell and a liquid/solid/hollow core. Typically, a biocompatible polymer shell of appropriate size and charge is used to protect the siRNA from degradation or sudden release and can be functionalized by modification with materials, such as PEG, PVA, polysaccharides, chitosan, and polyesters (PLA, PLGA, PCI) [127,128]. Although some limitations of polymer capsules have been reported, such as certain toxic effects, there are still many relevant and successful pieces of research [129]. Some scholars have reported a thermosensitive cationic polymer nanocapsule with a capsule shell consisting of Pluronic F127 and PEI. Pluronic F127 is a PEO-PPO-PEO non-ionic triblock copolymer composed of polyoxyethylene and polyoxypropylene ethers. The Pluronic-PEI capsule collapses at 37 °C and swells at 15 °C. After Pluronic-PEI siRNA are taken up by cells through endocytosis, the Pluronic-PEI volume rapidly expands following a 15 °C cold shock treatment. The process leads to Pluronic-PEI destroying the endosome and enabling siRNA to enter the cytoplasm. However, this technique currently can only be achieved in in vitro investigations [130]. Chen et al. [131] synthesized novel cationic NCs via the UV-induced thiolene interface crosslinking in transparent microemulsions, utilizing allyl-functionalized cationic polylactide (CPLA) as a raw material. Through electrostatic interaction, IL-8 siRNA binds to the cationic shell and encapsulates Dox in the inner cavity, allowing for co-delivery of the two drugs. CALP NCs have been demonstrated to be successful at resisting multidrug-resistant cancer cells and can be effectively taken up by cancer cells, showing a potential chemotherapeutic and gene silencing effect. New positively charged polymeric NCs derived from cucurbit[8]uril (CB[8]) and triviologen derivatives can adsorb siRNA with the advantages of high loading, high uptake and low toxicity, thereby protecting siRNA from degradation by enzymatic activity and allowing it to be released in acidic environments [132].

#### 5.2.3. Nanospheres

Different from the shell structure of polymer nanocapsules, polymer nanospheres (nanospheres, NPs) are compact polymer matrixes that can effectively load drugs [133]. Additionally, the ligand adsorbed on the particle surface can be targeted to a certain tissue or cell. Polymer materials are usually biodegradable, such as PLGA, chitosan, and PEI. PLGA nanospheres prepared by emulsion solvent diffusion have good safety and sustained release, making them good siRNA carriers. However, PLGA NPs normally are negatively charged, resulting in low transfection efficiency; after modification with chitosan, they can show a positive charge, which increases siRNA transfection and gene silencing efficiency [134]. Jensen et al. [135] manufactured a PLGA carrier incorporating siRNA for pulmonary drug delivery using the spray drying technique, thereby enabling lung-targeted drug administration without degrading siRNA activity. Inhaled PLGA nanoparticles reduce the number of doses administered and improve siRNA retention in the lung. In particular, when dipalmitoyl phosphatidylcholine (DPPC) and PLGA are used to form novel lipid-polymer nanoparticles as carriers for siRNA inhalation formulations, the composites increase the compatibility of the drug delivery system with the lung and the ability to penetrate the airway mucus layer and promote lung epithelial cell absorption without producing an inflammatory response [136].

Chitosan is biocompatible and can be employed independently as a nanodrug delivery system; however, its limited water solubility and poor cell permeability restrict its usage in siRNA drug delivery. Suhui et al. [137] adopted N,N,N-trimethyl chitosan (TMC), which has good water solubility and efficient transfection, as a polymeric carrier, and introduced trimeric phosphate (TPP) and baclofen (Bac) as an ionic cross-linker and carrier ligand, respectively. The final positively charged Bac-TMC and negatively charged TPP-Survivin siRNA formed Bac-TMC-TPP siSurvivin spontaneously under electrostatic interaction. The interaction of Bac-TMC with γ-aminobutyric acid β-receptor increased the uptake of siSurvivin. Encapsulated Bac-TMC-TPP siSurvivin nanoparticles were distributed in mannitol microparticles and dispersed in a pressurized metered dose inhaler (pMDI) device. The delivery method to the lungs demonstrated excellent aerodynamic properties and the ability to cross both lung and cellular barriers. Bac-TMC could be a novel carrier for siRNA inhalation preparations for lung cancer treatment.

#### 5.2.4. Dendrimers

Dendrimers are newly developed three-dimensional hyperbranched linear polymers comprising a central core unit, multiple layers of repeating branching units known as generations and terminal functional groups. They are spherical in solution and have the characteristics of multiple branch points, nano size, water solubility, biocompatibility, monodispersity and high drug loading capacity [138,139]. Common types of dendrimers are polypropyleneimine (PPI), polyamidoamine (PAMAM), polypeptides, etc. Dendrimers complexed with nucleic acids exhibit high transfection effectiveness, particularly those with an amine endgroup. Conti et al. [140] loaded siRNA onto positively charged fourth-generation PAMAM dendrimers (PAMAM G4NH2) and encapsulated the complexes in mannitol or chitosan-g-lactic acid (CSLA) and used hydrofluoroalkane (HFA) as the propellant to prepare pMDI. After long term exposure to HFA, the siRNA remained biologically active and the respirable particle index in the aerosol was as high as 77%, favoring deep lung deposition. Bielski et al. [141] modified PAMAM G4NH2 with TPP. The hydrophobic properties and spatial effects of the benzene ring in TPP reduce siRNA binding to the vector and facilitate siRNA release regulation. The efficacy of siRNA silencing genes in vitro was found to be greatest when the carrier contained 12 TPP molecules and the N/P ratio was 30. The transfection efficiency of siRNA was unaffected after being prepared into pMDI and DPI, and it was successfully transported to the lung, suggesting that it could be applied to treat lung-related disorders. However, dendrimers exhibit dose-dependent cytotoxicity, which is related to the number of end groups and surface charge. In general, positively charged dendrimers are more toxic than negatively charged and neutral dendrimers [138].

### 5.3. Pulmonary Surfactants

Although pulmonary surfactants (PSs) are a barrier to lung drug delivery and absorption, it has been demonstrated that using PSs in siRNA delivery vehicles can increase the solubility of insoluble drugs, enhance particle stability, and assist inhaled formulations in overcoming the lung barrier, optimizing drug delivery efficiency and minimizing side effects [60]. It has been demonstrated that PS-coated siRNA-containing nanogel carriers not only maintain the integrity of the particles but also have excellent pulmonary delivery capabilities after freeze-drying, reconstitution and nebulization by a vibrating mesh nebulizer without adding lyoprotectants [142]. According to the research, SP-B was discovered to enhance gene silencing effects by promoting siRNA delivery to the cytoplasm through electrostatic interaction with the anionic lipid membrane [143].

The characteristics of the vectors described above for the pulmonary delivery of siRNA are summarized in Table 4.

## 6. Clinical Application

Through continuous research on RNAi technology, a variety of siRNA drugs are currently being evaluated in clinical trials for the treatment of local or systemic diseases (Table 5), and four siRNA pharmaceuticals have been approved for marketing (Table 6) [5]. All of those approved siRNA pharmaceuticals are administered by injection. siRNA inhalation preparations have not yet been approved, but several drugs are in clinical trials.

ALN-RSV01, an siRNA inhalation preparation, was developed by Alnylam Pharms using RNAi technology to treat respiratory syncytial virus (RSV) infection. Except for two nucleotide overhangs at the 3′-end of the double strand, ALN-RSV01 has a total of 19 paired nucleotides without a chemical alteration or delivery system [144,145]. Intranasal spray targeting the mRNA of the RSV nucleocapsid protein (N protein) demonstrated antiviral activity against both RSV subtypes A and B [146]. ALN-RSV01 has undergone four clinical trials, with Phase I clinical trials in healthy adult men confirming its safety [146]. Then 88 healthy subjects were treated with ALN-RSV01 nasal spray or a placebo after being inoculated with RSV in a phase II clinical trial to determine whether ALN-RSV01 possessed antiviral activity. ALN-RSV01 was found to be capable of significantly reducing RSV activity [147]. Subsequent phase IIa and IIb clinical trials were conducted on patients with RSV infection combined with lung transplantation. However, no further progress has been made on the project as the final results did not reach the end point of the clinical trials [148,149]. Another siRNA inhalation formulation entering clinical trials is Excellair^TM^, developed by ZaBeCor Pharmaceuticals. It has no delivery vehicle, and targets spleen tyrosine kinase (Syk) mRNA to alleviate the inflammatory response and treat asthma. Phase I clinical trial results demonstrated that Excellair^TM^ was well tolerated by patients and significantly improved respiratory capacity in asthmatics with no significant adverse effects. Unfortunately, in 2015 the Phase II clinical trial of Excellair^TM^ was terminated for unknown reasons [7]. Besides, siRNA inhalation formulations are one possibility for effectively combating SARS-CoV-2 infections which are currently threatening human health. Research institutions and pharmaceutical companies worldwide are actively developing and screening siRNA drugs against the virus’s key genetic sequences [150]. The NRC Institute of Immunology FMBA of Russia [151] screened molecules from 15 anti-new coronavirus siRNAs and found that siR-7 was one of the most effective at inhibiting virus replication in vitro, and could target the virus-dependent RNA polymerase (RdRp). In order to enhance the in vivo stability and antiviral ability of siR-7, the investigators modified the structure by LNA at the 3′-end of the SS and AS of siR-7 to form siR-7-EM and then complexed it with the cationic peptide dendrimer KK-46 to finally prepare the siR-7-EM/KK-46 aerosol. Local administration of siR-7-EM/KK-46 to the lungs has been shown to effectively combat the SARS-CoV-2 and significantly reduce lung inflammation in preclinical trials. Currently, the Russian Federation’s Ministry of Health has approved the drug for clinical trials. Simultaneously, Alnylam Pharms and Vir Biotechnology collaborated to screen hundreds of siRNAs against highly conserved regions of the SARS-CoV-2 genome. VIR-2703 was found to have an EC50 < 100 pmol and an EC95 < 1 nmol in an in vitro viral model, making it one of the most effective drugs against SARS-CoV-2 to date. It could be used as an inhalation agent to prevent or treat the infection. The two companies intend to submit an IND application to the US FDA for VIR-2703 to treat COVID-19 [152].

**Table 5:** Representative clinical trials of RNAi therapeutics in the past 10 years.

Year	Therapeutic Name	Disease	Delivery Route	Phase Stage	Target	NCT ID	Ref.
2020	ALN-HSD	NASH	Subcutaneous	I	HSD17B13	NCT04565717	[153]
2020	DCR-PHXC	PH3	Subcutaneous	I	LDHA	NCT04555486	[154]
2019	Vutrisiran	ATTR With Cardiomyopathy	Subcutaneous	III	TTR	NCT04153149	[155]
2019	DCR-PHXC	PH1, PH2, Kidney Diseases	Subcutaneous	II	LDHA	NCT03847909	[154]
2018	Lumasiran	PH1	Subcutaneous	II	Glycolate oxidase	NCT03350451	[156]
2018	DCR-HBVS	Chronic Hepatitis B	Subcutaneous	I	HBV transcripts	NCT03772249	[157]
2018	siG12D-LODER	Pancreatic Cancer	Intravenous	II	KRAS G12D, all additional G12X mutations	NCT01676259	[6]
2018	Inclisiran	ACD	Intravenous	III	PCSK9	NCT03705234	[158]
2016	ARC-AAT	AATD	Intravenous	II	Z-AAT	NCT02900183	[159]
2016	ALN-HBV	HBV	Subcutaneous	I	HBV RNA	NCT02826018	[160]
2016	DCR-PH1	PH1	Intravenous	I	GO	NCT02795325	[161]
2016	ALN-TTRSC02	ATTR Amyloidosis	Subcutaneous	I	TTR	NCT02797847	[162]
2015	ARC-520	Chronic Hepatitis B	Intravenous	II	HBV DNA	NCT02349126	[163]
2015	ALN-CC5	PNH	Subcutaneous	I, II	C5	NCT02352493	[164]
2015	Fitusiran	Hemophilia A, B	Subcutaneous	I, II	AT	NCT02554773	[165]
2015	ALN-AS1	AIP	Subcutaneous	I	ALAS1	NCT02452372	[166]
2014	SYL040012	Open Angle Glaucoma	Ocular topical	II	ADR*β*2	NCT02250612	[68]
2014	ALN-TTR02	TTR-Mediated Amyloidosis	Intravenous	I	TTR	NCT02053454	[167]
2014	ALN-PCSSC	Hypercholesterolemia	Subcutaneous	I	PCSK9	NCT02314442	[168]
2013	ALN-TTRSC	TTR-Mediated Amyloidosis	Subcutaneous	II	TTR	NCT01981837	[169]
2012	SYL1001	Ocular Pain	Ocular topical	I, II	TRPV1	NCT01776658	[170]
2012	AVI-7100	Influenza	Intravenous	I	Influenza A M1/M2	NCT01747148	[171]

Notes: NASH, nonalcoholic steatohepatitis; PH3, primary hyperoxaluria type 3; ATTR, transthyretin amyloidosis; PH1, primary hyperoxaluria type 1; PH2, primary hyperoxaluria type 2; ACD; atherosclerotic cardiovascular disease; AATD, alpha-1 antitrypsin deficiency; PNH, paroxysmal nocturnal hemoglobinuria; AIP, acute intermittent porphyria.

**Table 6:** FDA-approved siRNA therapeutics.

Approval Date	Company	Therapeutic Name	Disease	Target	Delivery Route	Chemical Modification	Delivery System
August 2018	Alnylam	Patisiran	hATTR	TTR	Intravenous	2′-OMe modification	Second-generation LNPs
November 2019	Alnylam	Givosiran	AHP	ALAS1	Subcutaneous	PS linkages, 2′-OMe, 2′-F modification	GalNAc ligand conjugate
November 2020	Alnylam	Lumasiran	PH1	HAO1	Subcutaneous	PS linkages, 2′-OMe, 2′-F modification	GalNAc ligand conjugate
December 2020	Alnylam Novartis	Inclisiran	ACD	PCSK9	Subcutaneous	PS linkages, 2′-OMe, 2′-OMOE, 2′-F modification	GalNAc ligand conjugate

Note: Second-generation LNPs, containing CHOL, a polar lipid 1,2-distearoyl-sn-glycero-3-phosphocholine, PEGlated lipid PEG2000-C-DMG, ionizable amino lipid Dlin-MC3-DMA; AHP, acute hepatic porphyria; GalNAc, N-acetylgalactosamine.

## 7. Conclusions

siRNA inhalation formulations are an innovative and promising strategy for treating respiratory diseases. Although numerous researchers have conducted studies with favorable outcomes, no siRNA inhalation formulations have been clinically used to date. So why have the majority of positive results from in vitro and in vivo trials failed to cross over to clinical studies? There are two main reasons. First, as described in Section 3, inhaled drug delivery must overcome both a lung and a cellular barrier, whereas in vitro tests only require the cellular barrier to be overcome. Second, while the majority of in vivo studies of siRNA inhalation formulations have used rodents as models, the way experimental animals inhale drugs and the structural and physiological properties of their lungs are significantly different from those of humans. These differences may have contributed to the discrepancy in the results for rodents and humans.

Therefore, clinical applications need to be considered at the design and development stage for inhaled siRNA formulations. There are many factors affecting the efficacy of siRNA. The proper chemical modifications and efficient delivery vehicles become the key to enhancing the therapeutic effects of the drugs. However, although many siRNA modification methods have achieved promising results since the discovery of siRNA, there is still a lack of systematic research to demonstrate the benefits. In addition, high-efficiency and low-toxicity siRNA drug delivery vectors are also the focus of research. The application of non-viral vectors can greatly improve the delivery efficiency of inhaled siRNA formulations. Among the four siRNAs that have been marketed, three of them utilized the GalNAc-siRNA conjugate delivery system to achieve efficient liver-targeted delivery. Perhaps this could be used as inspiration to find the stable and efficient conjugated ligands that can be targeted to the lung. Furthermore, the choices of device and excipients for inhalation formulations are equally critical because they influence particle size distribution, spray pattern, drug loading, and stability, all of which, in turn, determine drug deposition in the lung. Nowadays, siRNA inhalation formulations are of increasing concern to mankind. Successful translation from basic research to the clinic remains a considerable challenge but the potential rewards are great.

## Figures and Tables

**Table 1:** Research of inhaled siRNA formulations for pulmonary cancer.

Disease	Target	Administration	Delivery System	Ref.
MDR-lung cancer	ABCC3	Inhalation	LPNs	[17]
Lung adenocarcinoma	VEGF	Inhalation	UCNP	[18]
Lung cancer	TUBB3	Oro-tracheal administration	NPs	[19]
Lung cancer	MRP1, BCL2	Inhalation	NLC	[20]
Lung cancer	Akt1	Inhalation	Nanosized polymer	[21]
Lung cancer	Mcl1	Intratracheal Instillation	Nanoliposomes	[22]
Lung metastasis	STAT3	Inhalation	PFC	[23]
NSCLC	EGFR-TKs	Inhalation	NLC	[15]
NSCLC	MRP1, BCL2	Inhalation	MSN	[24]

Notes: MDR, multidrug-resistant; LPNs, lipopolymeric nanoparticles; UCNP, up-conversion nanoparticle-based nanocage system; NPs, nanoparticles; NLC, Nanostructured Lipid Carriers; PFC, perfluorocarbon; MSN, mesoporous silica nanoparticles.

**Table 4:** The characteristics of the siRNA inhalation delivery systems.

Delivery Systems	Characteristics	
	Advantages	Disadvantages
Liposome	Improving siRNA stability, promoting endocytic uptake and transfection efficiency Facilitating siRNA endosomal escape	Prone to causing an inflammatory response Potentially cytotoxic
Solid lipid nanoparticle	High transfection rate Low cytotoxicity Prolonging in vivo half-lives of drugs	Low drug loading efficiency Poor stability
Nanostructured lipid carrier	Good bioavailability and biocompatibility Slow-controlled release capability High drug loading efficiency	Cytotoxic effects Irritating and sensitizing effects of surfactants
Nanoemulsion	Non-toxic and non-irritating Improving the stability and bioavailability of the drugs	Expensive preparation process Less selectivity of applicable excipients Instability during storage
Polymeric micelle	High structural stability and drug loading capacity Less immune response than liposomes Functional modification	Complex polymer synthesis Ineffective drug incorporation techniques
Polymer nanocapsule	Good biocompatibility Avoiding siRNA degradation and sudden release Functional modification	A certain toxic effect
Nanosphere	Biodegradable Sustained release Increasing the ability to penetrate the airway mucus layer	Low transfection efficiency of PLGA NPs Poor cell permeability of chitosan
Dendrimer	Multiple branch points Nano size Water solubility Biocompatibility Monodispersity High drug loading capacity	Dose-dependent cytotoxicity
Pulmonary surfactant	Increasing the solubility of insoluble drugs Enhancing drug stability Optimizing drug delivery efficiency	/
